# Multi-Scale Heart Beat Entropy Measures for Mental Workload Assessment of Ambulant Users

**DOI:** 10.3390/e21080783

**Published:** 2019-08-10

**Authors:** Abhishek Tiwari, Isabela Albuquerque, Mark Parent, Jean-François Gagnon, Daniel Lafond, Sébastien Tremblay, Tiago H. Falk

**Affiliations:** 1 Institut National de la Research Scientifique, Université du Québec, Montréal, QC H3A 0E7, Canada; 2 Thales Research and Technology, Québec, QC G1P 4P5, Canada; 3 School of Psychology, Université Laval, Québec, QC G1V 0A6, Canada

**Keywords:** mental workload, motif, multi-scale entropy, permutation entropy, HRV, SVM

## Abstract

Mental workload assessment is crucial in many real life applications which require constant attention and where imbalance of mental workload resources may cause safety hazards. As such, mental workload and its relationship with heart rate variability (HRV) have been well studied in the literature. However, the majority of the developed models have assumed individuals are not ambulant, thus bypassing the issue of movement-related electrocardiography (ECG) artifacts and changing heart beat dynamics due to physical activity. In this work, multi-scale features for mental workload assessment of ambulatory users is explored. ECG data was sampled from users while they performed different types and levels of physical activity while performing the multi-attribute test battery (MATB-II) task at varying difficulty levels. Proposed features are shown to outperform benchmark ones and further exhibit complementarity when used in combination. Indeed, results show gains over the benchmark HRV measures of 24.41% in accuracy and of 27.97% in F1 score can be achieved even at high activity levels.

## 1. Introduction

Mental workload is defined as the part of a person’s mental capacity that is needed to perform different demands brought on by a task [1]. Mental workload resources, if not used in a balanced way, can lead to a decrease in worker performance [2], as the resources are directly related to other mental concepts such as situational awareness, mental fatigue, and drowsiness, to name a few. In fact, in various jobs which require continuous attention for long durations of time, such as air traffic management, aircraft piloting, and emergency responders, overload of mental resources may lead to life-threatening outcomes. As such, mental workload assessment can be used to optimize operator performance by preventing the operators from becoming overwhelmed by the task at any point in time while also ensuring that they can perform the task efficiently. System design improvements can also be made to ensure balanced used of mental resources by proper assessment of mental workload. For example, car dashboards are designed in such a way that they provide the driver with all the necessary/critical information whilst minimizing mental workload. Similarly, aircraft cockpit design has been greatly simplified using mental workload considerations [3].

Mental workload is typically assessed using subjective, performance-related, and/or physiological methods. Subjective ratings are a direct way of mental workload assessment. Typically, such methods involve sampling the participant response to the amount of mental workload by using a questionnaire. The sampling can be done for every set amount of stimuli, or every set amount of time. Two of the most commonly used questionnaires are the (1) Subjective Workload Assessment Technique (SWAT) [4] and (2) NASA Task Load Index (NASA-TLX) [5]. The latter requires subjects to rate multiple dimensions that can then be aggregated into a single general workload index [6,7,8]. However, subjective ratings have some key limitations. First, they do not allow for continuous mental workload assessment and have poor temporal resolution. While increasing the sampling rate for operator feedback via the questionnaire could lead to improved temporal resolution, this may actually have negative impact of increasing the perceived workload due to the number of interruptions to the task being performed. Second, the ratings collected can be corrupted by subject bias, particularly if responses impact benefits received by the operators e.g., unpaid time off from work [6]. In turn, performance metrics related to the task can also be used to devise strategies for monitoring mental workload. Examples of such metrics include reaction time, number of errors, and task accuracy. However, one major barrier to applying these strategies to safety critical scenarios is the fact that the metrics can only be computed once the task is concluded.

In order to circumvent the limitations presented by these methods, operator physiological monitoring has emerged as a promising solution for mental workload assessment. Monitoring psycho-physiological signals allows for unbiased, continuous assessment of real-time mental workload while also being unobtrusive to the task at hand. In fact, with recent improvements in wearable technologies, remote monitoring of mental workload has been made possible even with operators in ambulatory conditions [9]. To this end, the electrocardiogram (ECG) has become an important modality to be measured. Heart rate variability (HRV), which is the variability in the inter-beat interval (RR) series derived from ECG, has shown to be an important correlate of psycho-social workload [10] (i.e., job stressors), mental workload and anxiety [11], as well as mental fatigue [12]. HRV is an indicator of the changes in the autonomic nervous system (ANS) and is controlled by both the sympathetic and para-sympathetic nervous system demands (increased activation of the sympathetic nervous system causes increases in heart rate, whereas an increased activation of the parasympathetic branch makes the heart rate slower). HRV has traditionally been quantified using time- and/or frequency-domain features computed from the RR time series [13]. The inter-beat interval series also exhibit complex fractal behavior with long-term correlations [14]. Over the last decade, these non-linear properties of the cardiac autonomic system have also been exploited using several complexity measures [15].

One such measure, called the multi-scale entropy (MSE) [16] has been proposed to characterize the complexity of physiologic time series at multiple scales. The algorithm is based on obtaining sample entropy at different time scales using a scaling algorithm. However, the originally proposed scaling algorithm (known as coarse graining) is sub-optimal and may lead to imprecise or undefined entropy values [17]. As a result, several variants of the multi-scale algorithm have been proposed in recent years [17], including permutation entropy (PE) [18,19] which has been shown to be robust to signal artifacts, as it deals with the shape of the time series, and not on magnitude values themselves [20,21]. Several variants of the coarse-graining method have also been proposed, including replacing it with moving average for short time series [22], a composite procedure that reduces the variance of entropy at higher scales [23], and the recently-proposed generalized multi-scale entropy measure [24], which quantifies the dynamics of the volatility (variance) of the time series over different scales [24,25].

Entropy-based measures have been used in the past to characterize aging and to diagnose different cardiac diseases [26,27,28]. Moreover, multi-scale analysis of the volatility series of the RR intervals [24] has also shown non-linear behavior and is able to successfully distinguish between healthy subjects and those with congestive heart failure. In [24], the authors argue that the coarse grained volatility series encapsulates additional information about the time series missed by the normal coarse graining procedure. Additionally the magnitude of the difference intervals of the RR series (i.e., $dRR_{i}=abs\left(RR_{i+1}-RR_{i}\right)$ has exhibited similar long-range correlations [29]. This property was used in [30] and showed better performance in distinguishing patients with congestive heart failure at lower scales compared to the RR series. Further, several improvements to the permutation entropy measure have been proposed. The modified permutation entropy (mPE), for example, takes into account cases in which instantaneous heart rate measures remain the same for two consecutive beats [31], while the weighted permutation entropy [32] tries to incorporate the amplitude information of the time series being analyzed.

While such multi-scale measures have been used in cardiac disease monitoring, they have received little attention for mental state monitoring. However, PE was recently used for emotion assessment [33] of stationary users. Here, we are particularly interested in assessing user mental states in an ambulatory setting, in which movement may not only introduce artifacts that play a detrimental role in signal quality, but also cause changes in cardiac dynamics that may alter HRV measurement. As such, we explore a number of existing multi-scale features and propose new ones for mental workload assessment as subjects performed two different physical activities at three different levels. We hypothesize that the noise robustness provided by the permutation entropy measure coupled with studying complexity at different scales would help better quantify heart rate changes due to mental workload at different levels of physical activity.

The remainder of this paper is organized as follows. Section 2 describes the materials and methods used, including the database considered, proposed and benchmark features, prediction method, and performance metrics used. Section 3 then presents and discusses the results obtained and conclusions are presented in Section 4.

## 2. Materials and Methods

Here, we describe the database used, benchmark features, the different multi-scale methods tested, as well as the feature selection scheme employed, classifiers, and analysis pipeline.

### 2.1. Data Collection

#### 2.1.1. Participants

After screening, 47 participants were selected (23 female, 27.4±6.6 years old). Screening was performed in order to prevent any potential risk to the participants during the experiments. Candidates with cardiovascular diseases, neurological disorders, history of feeling dizzy or fainting were excluded from the experiment. The participants were asked to wear comfortable sportswear. Twenty-two participants utilized a treadmill during the experiment and 26 a stationary bike. Participants consented to participating in the experiment and were remunerated for their time. The experimental protocol was approved by the Ethics Review Boards of INRS, Université Laval and the PERFORM Centre (Concordia University), the latter being the location in which data was collected.

#### 2.1.2. Experimental Protocol

**Figure 1:**
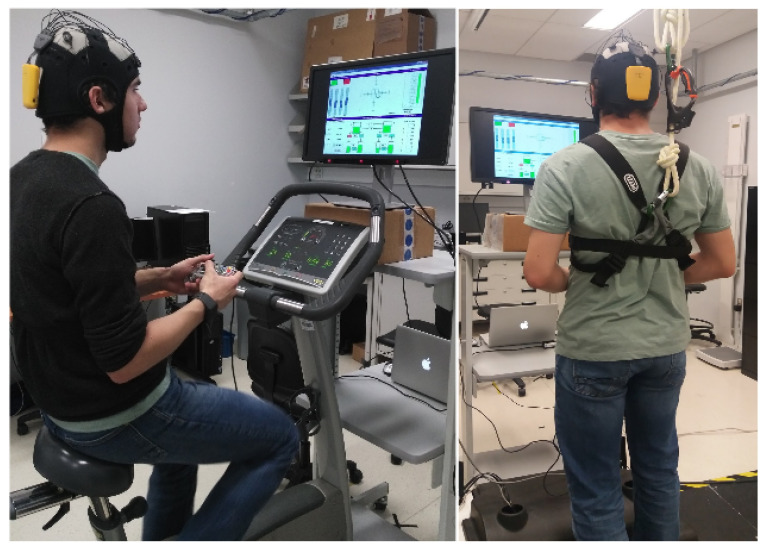
Experimental setup for both bike and treadmill conditions.

Before starting the data collection, a tutorial explaining the experimental procedure and task to be executed was shown to the participants. Next, various sensors were placed on the subject. These included a portable eight-channel wireless EEG headset (Enobio, Neuroelectrics), a portable chest strap (Bioharness 3, Zephyr) for ECG, respiration, and accelerometer signal recording, an E4 (Empatica) wrist watch which measures blood volume pulse, skin temperature, galvanic skin response and acceleration. Following this step, in the case of participants using the treadmill, a safety harness was placed on the participant’s chest to avoid falls. For participants using the stationary bike, they were asked to adjust the seat according to their preference. The height of the screen was then adjusted to provide a more comfortable set-up. Three levels of physical activity were considered: no movement, medium (treadmill: 3 km/h, bike: 50 rpm), and high (treadmill: 5 km/h, bike: 70 rpm). Figure 1 shows the experimental setup for both bike and treadmill conditions.

**Figure 2:**
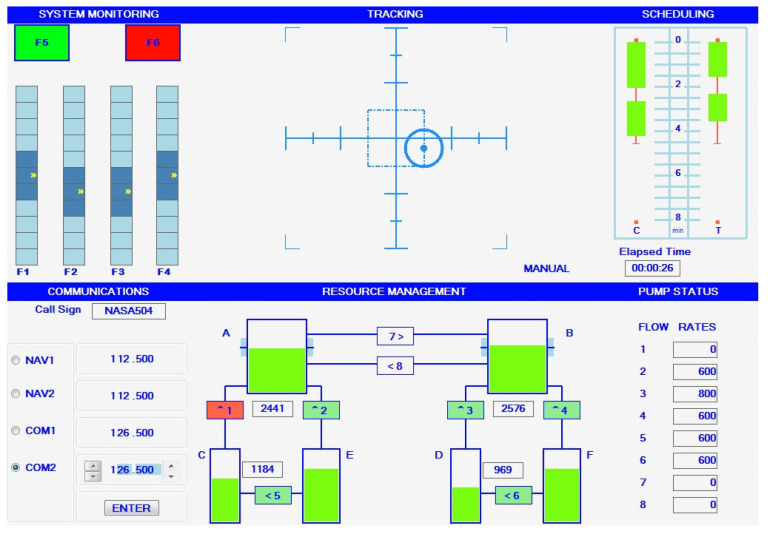
Multi-attribute task battery (MATB-II) game for eliciting different levels of mental workload.

In order to elicit high and low mental workload levels, the NASA multi-attribute task battery (MATB-II) was used. It is a computer-based task designed to evaluate operator performance and mental workload. Figure 2 depicts the MATB-II interface seen by the participant [34]. MATB-II encompass four tasks, all grouped under a single-window interface. These four tasks are the system monitoring (top-left), the tracking (top-center), the communications (bottom-left) and the resource management (bottom-center). In this study, the communication task was not used. The interface also includes a scheduler (top-right), which only showed the time remaining in each trials, as well as the pump status (bottom-right), which complemented information on the resource management task (see below). Since participants were simultaneously doing a physical task, a mouse could not be used to interact with MATB-II. Instead, participants were instructed to use an xBox One controller.

The system monitoring task requires the participant to monitor four sliders and report deviations from their normal state. The two warning lights (see F5 and F6 on Figure 2) were not used in this study. In their normal states, sliders were oscillating around the middle position. In their deviation state, sliders started oscillating around the top or the bottom of the sliders. Participant had to use the directional pad of the controller to report deviations. The tracking task requires the participant to keep a target (the circle) within a box (the square). As the trials went on, the target started to move randomly. Participants had to use the joystick of their controller to brink it back near the center of the square. The resource management task requires the participant to balance a network of fuel tanks. Participants were instructed to keep the level of tanks A and B as close as possible from 2500 units (this level is indicated by ticks on tanks A and B, see Figure 2). Fuel gradually depleted from tanks A and B. To keep the tanks at level, participants could use eight pumps (labeled 1–8, between tank) to move fuel between tanks. To activate pumps, participants had to use the other joystick of the controller to move the cursor and “click” on the pumps. Pumps were configured to fail from times to times. When a pump failed, it turned red and became unusable. Pumps were “repaired” automatically after a while and the participant could resume using it if needed.

Two levels of workload were used for the mental task. Compared to the low workload condition, the high workload condition had: more frequent sliders deviations (for system monitoring), faster random oscillations (for the tracking task) and more frequent pump failures (for resource management monitoring).

In total, six combinations of combined mental workload and physical activity were tested (two level of mental workload X three levels of physical activity). The experiment was then split into six sessions, each one corresponding to one of the six combinations previously described. The order in which the combinations were done was counterbalanced to avoid ordering effects. Before each session, two baseline sessions were performed. During the first session, there were neither physical or mental activity and the participants were asked to close their eyes and relax for 60 s. Then the subject was asked to open their eyes and start moving according to the corresponding sessions physical activity until reaching the desired level. After reaching a stable activity level, the second baseline was collected while the participant kept the pace for 2 min without any mental effort involved. This baseline has been added to ensure the stationarity of the heart rate dynamics for a given activity level prior to introducing the mental workload condition. This Lastly, the experimenter gave the joystick to the participant who then started the first experimental session for a duration of 10 min. After each session, a 5-min break was given. After each of the experimental sessions, participants were asked to fill the NASA-TLX questionnaire and report their perceived fatigue level based on the Borg scale. The NASA-TLX ratings were validated with respect to the ground truth in [9] Overall, the experimental protocol lasted roughly two hours.

### 2.2. Pre-Processing

In this study, we focused only on the ECG signal measured by the Bioharness Bh3 device, sampled at 250 Hz. This sampling rate allowed for continuous streaming throughout the experiment without the need to recharge the device. Such sampling rate has been successfully used in a number of different applications, including [35,36,37]. First, the ECG signal for all subjects was visually inspected and two subjects were removed as the data was corrupted due to sensor malfunction. For the remaining subjects the inter-beat interval series was extracted as follows. First, the ECG was filtered using a band-pass filter with a bandwidth 4–40 Hz to enhance the QRS complex. This was followed by an energy based QRS detection algorithm [38], which is an adaption of the popular Pan and Tompkins algorithm [39]. The RR series was further filtered to remove outliers using range based detection (≥280 ms and ≤1500 ms), moving average outlier detection, and a filter based on percent change in consecutive RR values (≤20%) as implemented in [40].

### 2.3. Benchmark HRV Features

Standard time- and frequency-domain HRV metrics were extracted and used as benchmark measures. A complete list of these conventional measures can be found in Table 1. The majority of these benchmark features have been shown in the literature to correlate with mental workload [41] and anxiety [11]. Complete details about these measures can be found in [13]. A total of 15 benchmark features were extracted over 5-min segments of RR series with a 4-min overlap, resulting in six RR series for each of the 10-min experimental sessions. The 5-min windows for HRV analysis follows recommendations from [13]. Notwithstanding, shorter time series may cause problems with multi-scale entropy estimation. In order to overcome this limitation, focus has been placed on multi-scale entropy methods designed specifically for short term analysis of HRV recordings [22,23].

### 2.4. Multi-Scale Entropy Features

The multi-scale entropy methods rely on two steps: (i) scaling and (ii) entropy calculation over the different scales. Here, we explored different algorithms for both steps, as summarized in Table 2.

#### 2.4.1. Scaling Algorithms

Several scaling algorithms have been proposed in the literature and attempt to convey fractal information at different scales. All of these methods take the original time series (x(i)) with an index *i* and produce the time series for a different scale. Details about the methods explored herein are given next.

**Figure 3:**
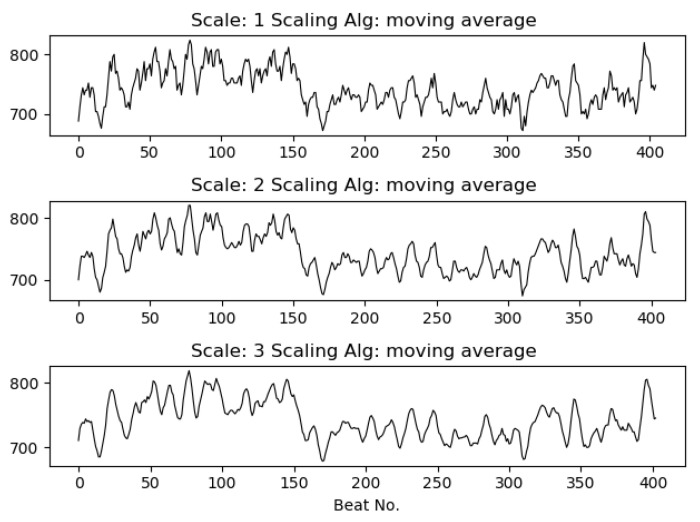
Scaled inter-beat interval (RR) time series with the moving average algorithm for scales s=1 (original series) to s=3.

**Figure 4:**
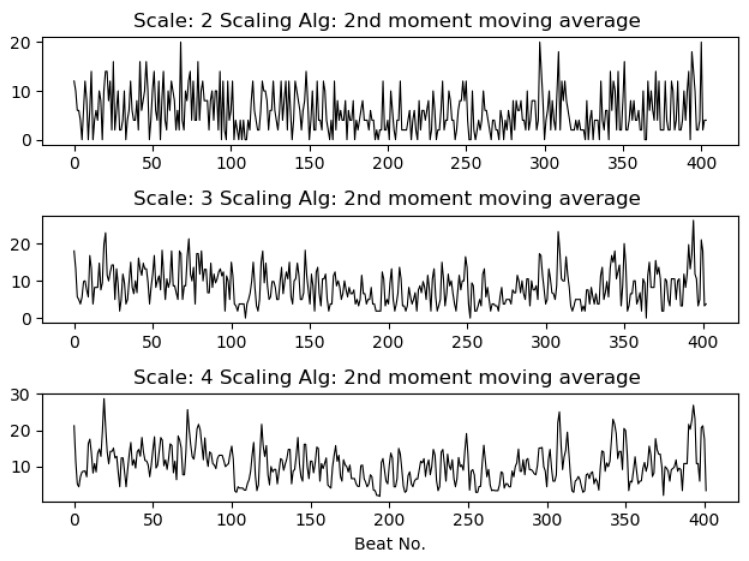
Scaled RR time series with the second moment moving average algorithm for scales s=2 to s=4.

- Coarse graining (cg): This is the original algorithm proposed to obtain different scales. A point *j* on the scaled series $y_{s}\left(j\right)$ for a scale *s* is given by:
  (1) $$y_{s}\left(j\right)=\frac{\sum_{i=(j-1)s+1}^{js}x\left(i\right)}{s},$$
  where, 1≤j≤N/s. This method has been shown to be sub-optimal [17] and to lead to scaled series that decrease in size, which could increase the variance in the estimated entropy [23]. As a result, several variants have since been proposed, including the remainder listed below.
- Moving average (mavg): With moving average, a point *j* on the scaled series $y_{s}\left(j\right)$ for a scale *s* is given by:
  (2) $$y_{s}\left(j\right)=\frac{\sum_{i=j}^{i=j+s-1}x\left(i\right)}{s},$$
  where 1≤j≤N−s+1.
- Composite coarse graining (comp_cg): the composite method generates *s* different (from k=1,…,s) scaled series for a given scale *s*. The entropy estimates from the different series for the scale *s* are then averaged to get the entropy estimate. This helps reduce the error in the entropy estimation that occurs due to coarse graining produce. For a given scale *s* the point *j* of the $k^{th}$ coarse grained series ($y_{k,s}\left(j\right)$) is given by:
  (3) $$y_{k,s}\left(j\right)=\frac{\sum_{i=(j-1)s+k}^{js+k-1}x\left(i\right)}{s},$$
  where 1≤j≤N/s, and 1≤k≤s gives the next index of the scaled series. The composite multi-scale entropy (CMSE) for a given scale is then given by:
  (4) $$CMSE\left(s\right)=\frac{\sum_{k=1}^{s}Ent\left(y_{k,s}\right)}{s},$$
  where Ent is the entropy calculation algorithm. In the original CMSE algorithm the sample entropy algorithm is used.
- The second moment coarse graining (mom): this method quantifies the standard deviation of the scaled series (also called the volatility series) rather than its mean as done by the coarse graining procedure. A point *i* on the scaled series $y_{s}\left(i\right)$ for a scale *s* is given by:
  (5) $$y_{s}{\left(j\right)=\sigma |}_{i=(j-1)s+1}^{js}\left(x\left(i\right)\right),$$
  where 1≤j≤N/s and σ represents the standard deviation.
- The second moment moving average (mavg_mom): we propose the moving average procedure for calculation of the second moment to adapt to short time series. This replaces the non-overlapping windows used in coarse graining to a sliding window, as in the case of the moving average algorithm.
  Figure 3 and Figure 4 show the RR series and RR volatility series (using mavg_mom) for scales s=1 to s=3 and scales s=2 to s=4, respectively, for a five minute ECG segment. As can be seen, scaling removes some high frequency information from the series, commonly associated with artifacts.

#### 2.4.2. Entropy Algorithms

**Figure 5:**
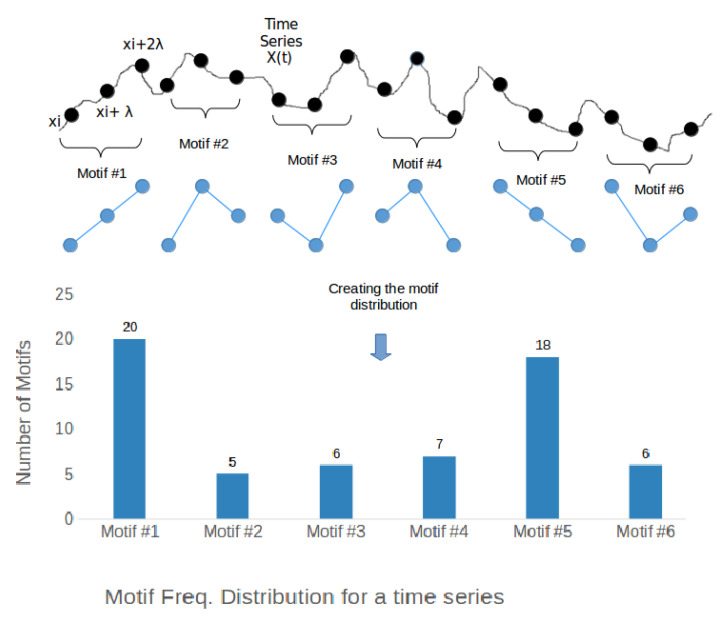
Original motifs of degree m=3 appearing in a time series.

- Sample entropy (SampEn): it is the negative natural logarithm of an estimate of the conditional probability that if two sets of vectors ($X_{m}\left(i\right)$ and $X_{m}\left(j\right)$) of length *m* have a distance <r, then two sets of vectors ($X_{m+1}\left(i\right)$ and $X_{m+1}\left(j\right)$) of length m+1 also have a distance <r, based on some distance metric $d_{m}\left(X,Y\right)$. It is formally defined as:
  (6) $$SampEn=-\log\frac{N_{m+1}}{N_{m}},$$
  where $N_{m}$ is number of vector pairs with $d_{m}\left(X_{m}\left(i\right),X_{m}\left(j\right)\right)<r$ and $N_{m+1}$ is number of vector pairs with $d_{m}\left(X_{m+1}\left(i\right),X_{m+1}\left(j\right)\right)<r$.
- Modified permutation entropy (mPE): the permutation entropy algorithm quantifies the occurrence of motifs in the series. Motifs are defined as recurring patterns in the time series with a degree *m* and lag λ. Based on the rank ordering of the motif pattern we assign it a specific symbol *j*. Representative motifs of degree (m=3) and lag (λ=1) are shown in Figure 5. For modified permutation entropy, to account for stationary consecutive beats, four additional symbolic representations have been added (from 7 to 10 as shown in the equation below). The time series (X(t)) is first converted to the ordinal series ($X^{m,\lambda }\left(j\right)$), where 1≥j≤N−m where *N* is the size of the time series using the following relations:
  $$X^{m,\lambda }\left(j\right)=\left\{\begin{array}{cc} 1 & \mathrm{if}\;X(i)<X(i+\lambda )\;\&\;X(i+\lambda )<X(i+2\lambda )\;\&\;X(i)<X(i+2\lambda )\\ 2 & \mathrm{if}\;X(i)<X(i+\lambda )\;\&\;X(i+\lambda )>X(i+2\lambda )\;\&\;X(i)<X(i+2\lambda ),\\ 3 & \mathrm{if}\;X(i)>X(i+\lambda )\;\&\;X(i+\lambda )<X(i+2\lambda )\;\&\;X(i)<X(i+2\lambda ),\\ 4 & \mathrm{if}\;X(i)<X(i+\lambda )\;\&\;X(i+\lambda )>X(i+2\lambda )\;\&\;X(i)>X(i+2\lambda ),\\ 5 & \mathrm{if}\;X(i)>X(i+\lambda )\;\&\;X(i+\lambda )>X(i+2\lambda )\;\&\;X(i)>X(i+2\lambda ),\\ 6 & \mathrm{if}\;X(i)>X(i+\lambda )\;\&\;X(i+\lambda )<X(i+2\lambda )\;\&\;X(i)>X(i+2\lambda ).\\ 7 & \mathrm{if}\;X(i)==X(i+\lambda )\;\&\;X(i+\lambda )<X(i+2\lambda ).\\ 8 & \mathrm{if}\;X(i)==X(i+\lambda )\;\&\;X(i+\lambda )>X(i+2\lambda ).\\ 9 & \mathrm{if}\;X(i)>X(i+\lambda )\;\&\;X(i+\lambda )==X(i+2\lambda ).\\ 10 & \mathrm{if}\;X(i)<X(i+\lambda )\;\&\;X(i+\lambda )==X(i+2\lambda ). \end{array}\right.$$
  The modified permutation entropy (mPE) is then given by:
  (7) $$mPE=-\sum_{j}^{m!+n}p\left(\pi_{j}^{m,\lambda }\right)\cdot \log\left(p\left(\pi_{j}^{m,\lambda }\right)\right),$$
  where *n* is the number of additional motif patterns added for the modified permutation entropy and $p(\pi_{j}^{m,\lambda })$ is the relative frequency of the motif pattern represented by $\pi_{j}^{m,\lambda }$ and calculated as:
  (8) p(πjm,λ)=∑j≤m!+n╟u:type(u)=πj(Xjm,λ)∑j≤m!+n╟u:type(u)∈Π(Xjm,λ),
  where ╟A(u) denotes the indicator function of set A defined as ╟A(u)=1 if u∈A and ╟A(u)=0 otherwise and type(.) denotes the map from pattern space to symbol space.
- Weighted modified permutation entropy (mPE_wt): weighted permutation entropy was proposed to incorporate the amplitude information into the permutation entropy algorithm. This is done by calculating the variances (referred as weights $w_{i}$) corresponding to each motif pattern in the time series. The relative frequency $p(\pi_{i}^{m,\lambda })$ of a given pattern is then calculated as:
  (9) p(πjm,λ)=∑j≤m!+n╟u:type(u)=πi(Xjm,λ)wj∑j≤m!+n╟u:type(u)∈Π(Xjm,λ)wj.
  The permutation entropy for this adjusted relative frequency is then calculated using the standard permutation entropy equation in (8).

The entropy values were calculated for both the RR series and the dRR series for a scale of s=1,…,10. Moreover, the mean and standard deviation of the entropy measures across all scales were calculated, thus resulting in 24 total features for each type of entropy measure.

#### 2.4.3. Ordinal Distance Dissimilarity

Ordinal distance based dissimilarity [42] can be used to calculate the difference between the two ordinal series. The distance between two ordinal series *X* and *Y* is given by

(10) $$D_{m}\left(X,Y\right)=\sqrt[{}]{\frac{m!}{m!-1}}\sqrt[{}]{\sum_{j}^{m!}{\left(p_{x}\left(\pi_{j}^{m,\lambda }\right)-p_{y}\left(\pi_{j}^{m,\lambda }\right)\right)}^{2}},$$

where $p_{x}\left(\pi^{m,\lambda }\right)$ and $p_{y}\left(\pi^{m,\lambda }\right)$ are the relative frequencies of the motif pattern represented by $\pi^{m,\lambda }$ in series X and Y, respectively, and m is the degree of the motif. As the RR series has statistical fractal properties (the statistical properties over different scales do not change), the ordinal distance has been calculated over the different scaled series (referred to as the inter-scale ordinal distance ($isod_{s_{x},s_{y}}$)). To limit the feature space size, we have calculated the distances between scales $s_{x}=1\;to\;s_{x}=3$ and $s_{y}=1\;to\;s_{y}=10$ with $s_{x}\neq s_{y}$. This was done for both the RR and dRR series. Additionally, we calculate the statistics of isod for given $s_{x}=1$ to $\;s_{x}=3$ relative to all $s_{y}$, we calculate the mean, standard deviation and first difference of $isod_{s_{x}}$, thus resulting in a total number of ordinal distance features of 66. For simplicity, only the original permutation entropy based motif structures are considered and the scaling is done by the moving average scaling algorithm.

### 2.5. Feature Selection and Classification

For evaluation, a five-fold cross validation setup was used. Workload assessment was performed as a binary classification task, where the high and low mental workload ground truth labels are taken from the from the MATB-II task. A support vector machine (SVM) classifier with an RBF kernel was used. To explore the generalization performance, the above mentioned procedure is repeated 50 times with different random seeds. This leads to 250 (5-folds times 50 repetitions with different random seeds) training and test sets and classifications. To assess feature importance, we used feature selection and look at the frequency of features occurring in the top 20 sets for the 250 possible combinations.

To assess feature importance, recursive feature elimination was performed using the extra trees classifier [43]. Given an external estimator that assigns weights to features (an extra trees classifier in this case), the least important features are pruned from the current set of features. The procedure is recursively repeated on the pruned set until the desired number of features to be selected is reached. This technique considers the interaction of features with the learning algorithm to give the optimal subset of features. The feature selection is used to select the top 20 features for each fold of the cross-validation set. The implementation of the classifier and feature selection algorithms was done using sci-kit learn [44].

## 3. Results and Discussion

The performance of the different entropy and scaling methods, the inter-scale ordinal distance and benchmark features were compared for mental workload assessment. Comparison was done for different levels of physical workload, thus allowing for the robustness of the features to be assessed relative to increases in movement artifacts and changing dynamics of the heart rate brought on by physical activity. We first compared the performance of the different combinations of entropy and scaling approaches for different activity levels. The best performing algorithms were then compared to the performance of ordinal distance scale similarity measure and benchmark features. Additionally, we performed feature fusion where all the different feature sets were combined to test for feature set complementarity.

### 3.1. Comparing Different Multi-Scale Entropy Algorithms

**Figure 6:**
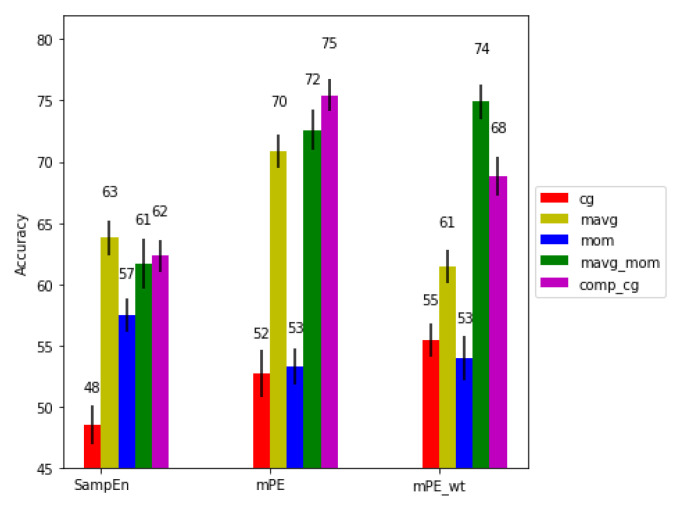
Performance comparison for the no physical activity condition.

**Figure 7:**
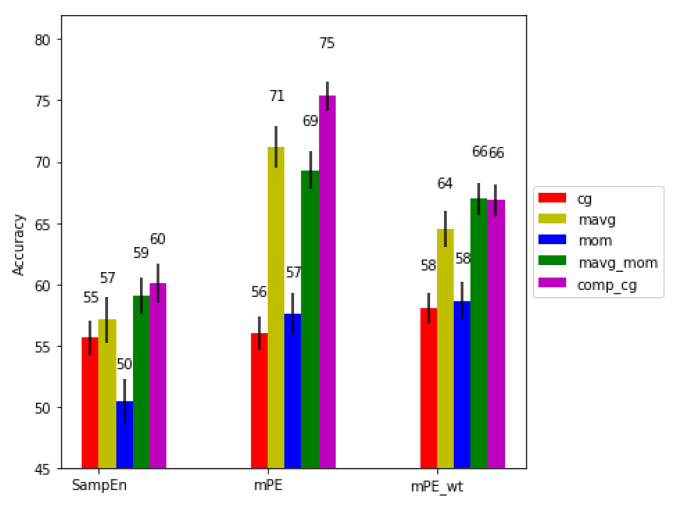
Performance comparison for the medium physical activity condition.

**Figure 8:**
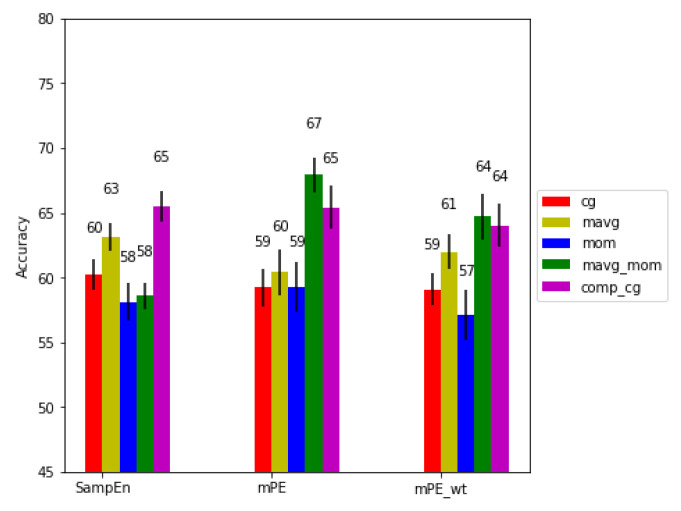
Performance comparison for the high physical activity condition.

We calculated the accuracy (Acc) for different activity levels with the combinations of the three entropy measures and five scaling algorithms. Figure 6, Figure 7 and Figure 8 show the performance of the algorithms for no, medium and high physical activity levels, respectively. As can be seen, generally across all physical activity cases and for all entropy algorithms, the short time moving average based scaling (mov_avg and mavg_mom) and composite based scaling (comp_cg) methods outperform coarse graining based approaches (cg, mom). It can also be seen that the modified permutation entropy based on second moment based scaling methods (mom and mavg_mom) (referred to as generalized permutation entropy in [24]) typically achieve higher predictive power across all physical workload cases, hence indicating the importance of volatility series of the RR series, as well as dRR series. Lastly, the modified permutation entropy based algorithms (mPE and mPE_wt) performs better than sample entropy based methods.

Specifically, looking at the performance of the features across different physical activity level conditions, it can be seen for the no physical activity condition that mPE with (comp_cg and $mavg_{m}om$) achieved significantly higher performance (p<0.01) than most of the other methods. Moreover, by including the amplitude information into the mPE via the $mPE_{wt}$ measure, a drop in performance is seen for the moving average scaled RR series, but the best performance for the volatility scaled series is achieved with our proposed moving average scaling of the second moment (mavg_mom). These results suggest that amplitude information of the volatility series is important for mental workload assessment.

For the medium physical activity level condition, we observe similar performance trends to the no physical activity condition with mPE (comp_cg scaling) achieving significantly higher performance (p<0.01) than the other methods. Interestingly, incorporating the amplitude information in this condition leads to a decrease in performance for both comp_cg and mavg_mom. This could be due to the changing cardiac dynamics during physical activity.

Finally, for the high physical activity level condition, we see an overall drop in performance compared to the other two physical activity levels. We observe that the SampEn performance is comparable to mPE and $mPE_{wt}$ for certain scaling methods (cg, mavg and comp_cg). We also see a drop in performance for mPE when using mavg compared to cg scaling, though this is not observed for SampEn and mPE_wt, both of which show improvement on using the mavg scaling. Overall, we achieve the best performance using the modified permutation entropy for proposed short time second moment calculation and comp_cg, using the mPE, i.e., excluding the amplitude information. Higher performance without incorporating amplitude information (not using $mPE_{wt}$) in both medium and high physical activity level conditions could be due to higher noise in the RR series arising from misdetections in the QRS complex caused by movement artifacts. As $mPE_{wt}$ is sensitive to such artifacts, ignoring the amplitude information is better in such cases. Overall, mPE with comp_cg and mavg_mom achieved the best results, with mavg_mom based scaling giving significantly higher results than all the other methods tested (p<0.01).

Moreover, as [24] emphasizes the complementary nature of the volatility series, we further investigate the fusion of comp_cg and mavg_mom base scaling methods using the mPE algorithm, as these resulted in consistently better performance across all three physical activity conditions. Table 3 shows the results of fusion for the different physical activity levels. As can be seen, for the no and medium physical activity levels, fusion gave a significant (p<0.01) improvement of 3.53% in accuracy and 3.30% in f1-score and 1.90% accuracy and 1.63% f1-score, respectively, over the best performing comp_cg + mPE algorithm. However, no improvement was seen for high physical activity level. Such findings corroborate those of [24].

### 3.2. Gauging Performance Against the Benchmark

Here, we compare the performance of the best performing algorithms from Section 3.1 with the benchmark features. We further perform feature fusion of the two sets to further explore their complementary. Table 4, Table 5 and Table 6 shows the benchmark, inter-scale ordinal distance, and best performing multi-scale entropy methods and their fusion for no, medium and high physical activity levels, respectively. In the tables, ‘nof’ indicates number of features used in each case.

As can be seen, for the no physical activity condition, both multi-scale entropy and the inter-scale ordinal distance features perform significantly better than the benchmark with improvements of 21.21% in accuracy and 28.25% in f1-score and 20.66% in accuracy and 28.91% in f1-score, respectively. Additionally, fusion provides significant (p<0.01) improvements of 5.45% accuracy and 5.42% f1-score over the multi-scale features alone.

Similarly for medium physical activity levels, both multi-scale entropy and the inter-scale ordinal distance features performed significantly better than the benchmark with improvements of 24.08% in accuracy and 16.82% in f1-score and 27.20% in accuracy and 21.84% in f1-score, respectively. In this case, the inter-scale ordinal distance features performed significantly better (p<0.01) than the multi-scale entropy features. Fusion also improved performance significantly (p<0.01) and results in gains of 2.12% accuracy and 2.07% f1-score over the inter-scale ordinal distance features. Lastly, for the high physical activity level condition, both multi-scale entropy and the inter-scale ordinal distance features performed significantly better than the benchmark with improvements of 9.90% in accuracy and 13.05% in f1-score and 20.74% in accuracy and 24.25% in f1-score, respectively. Again, the inter-scale ordinal distance features performed significantly better (p<0.01) than the multi-scale entropy features. Additionally, fusion gave a significant (p<0.05) improvement of 1.90% accuracy and 1.69% f1-score over the inter-scale ordinal distance features. The improvement in performance achieved with fusion for all three activity levels further corroborates the results of [24].

### 3.3. Feature Ranking

Feature importance was computed based on the outcomes of feature selection across the five cross validation steps, repeated 50 times. The top 20 features were selected for every fold. As such, the frequency of occurrence of a given feature in the top feature set was calculated over the 250 iterations. Features appearing more than 70% were further ranked according to their frequency of occurrence (freq) for no, medium and high physical activity levels. These values are reported in Table 7, Table 8 and Table 9, respectively, along with the feature names.

As can be seen, for the no physical activity condition, we observe that three of the eight top-ranked features are from the inter-scale ordinal distance feature set with interaction of different scales with s=3 being the case for two of the three isod features. Additionally, one multi-scale mPE features show up in the most frequent set with composite scaling based entropy of s=3, Additionally, we see four benchmark features in the top feature set, with three statistical features as well as the ratio of low to high frequency (lf/hf). A consistent decrease in the mean of RR has been reported in the literature with increased mental stress, a similar trend in the standard deviation of RR intervals could explain the overall importance of the coefficient of variation which is a ratio of the two [41]. Similarly, an increase in the lf/hf ratio was reported for increased mental workload across various studies [41]. We also observed that one of the proposed feature was calculated over the dRR series (isod feature), reflecting the presence of long-term correlations and complexity in the magnitude difference of RR series as noted in [29]. The presence of the different feature sets along with the benchmark features further corroborates the complementary nature of the features.

Similarly, looking at medium physical activity level conditions, we observe that of the 12 most frequent features, five were from inter-scale ordinal distance features with interactions between s=1 and s=2 with other scales. Further, additionally six multi-scale mPE features showed up in the most frequent set with composite scaling based entropy of the original time series (s=1) (same as for mov_mom with s=1 as well), along with s=9 and s=10 as well as of second moment from s=5 and s=9. Additionally, scales s=8 for the mPE of second moment also shows up in the top features for both the RR and dRR series. We only have one benchmark feature (mean RR) in this case among the top features, thus suggesting their sensitivity to movement artifacts. One of features were calculated on the dRR series.

Lastly, for the high physical activity level conditions, of the top 10 most occurring features, we observed that two features are from the inter-scale ordinal distance features, five features are from the multi-scale mPE entropy with composite scaling based entropy of the scales s=2, s=4, s=6 and s=8 as well as of second moment from s=3. Interestingly, no entropy feature from the original time series (s=1) was seen in the top features. We also observed three benchmark features in the top set, with both normalized low and high frequency along with mean of absolute first difference.

Mental workload has reported a drop in HRV features [45,46] attributed to sympathetic activation and/or para-sympathetic withdrawal [45,46,47,48]. Time and frequency domain HRV features were focused on characterizing the balance between these two systems. However, a lack of clear unbalance of the ANS due to mental workload has been reported in the literature [49]. This has shifted focus on the use of non-linear descriptors based on complex systems approach to better characterize the fractal RR time series [15]. These methods often characterize the complexity of the RR time series [50]. Recent studies indicate that this complexity is a result of both sympathetic and parasympathetic components of the ANS [51]. A recent study [49] has shown correlation dimension, which measures the fractal self-similarity of signal, decrease to comparable pathological values during mental workload inducing tasks, which indicates an suppression of the parasympathetic activity in the heart [52] and breakdown of long term correlations in the RR series [14] which can be quantified by complexity at higher scales [26].

A few studies have looked at the effects of exercise on HRV features. [53] reported an increase in overall complexity due to walking (4 km/hr) along with a significant increase in normalized low frequency power and a decrease in normalized high frequency power. A similar trend for low intensity exercise was reported in [54] with a contradictory increase in the high frequency component with increased exercise intensity on a bicycle. This increase has further been explained by the influence of breathing on heart rate (respiratory sinus arrhythmia (RSA)) which has a strong high frequency component during high intensity exercise [55]. Interestingly, when looking at the non-linear properties of the heart rate for high intensity exercise, entropy (scale=1) decreases while complexity is still retained at different scales [56], something that can be exploited by multi-scale entropy measure.

The scaling process for the multi-scale entropy algorithm is equivalent to low pass filtered frequency bands with decreasing bandwidth with increasing scales [57]. This scaling can be achieved by different types of scaling operations. For our study we have focused on two methods, namely composite coarse graining and moving average scaling methods. Given the presence of two distinct frequency regions in the heart rate due to parasympathetic activity (corresponding to high frequency fluctuations in the RR series) and sympathetic activity (corresponding to lower frequency fluctuations) [13], the multiscale entropy algorithm represents the complexity of the overall series due to interaction of both sympathetic and parasympathetic systems at lower scales, while representing the complexity of lower frequency component (mostly due to sympathetic activity) at higher scales. Furthermore, the inter-scale ordinal distance feature tries to quantify the complex interaction between the different frequency regions.

In keeping with the above variations in ANS balance with mental workload and exercise we observe a scale s=3 show importance for no physical workload case which captures more lower frequency information compared to original scale. With medium physical activity further contributing to the increase lower frequency components in mental workload we observe entropy of higher scale of s=8 to s=10 (capturing low frequency information) show up in the top feature sets. Finally for high physical activity where high frequency components show important contribution due to the influence of RSA to the heart rate we see both low (s=2 and s=3) and high (s=4, s=6 and s=8) scales for entropy show significance in the top feature set. Additionally we see the normalized high and low frequency components among the top features which show significance during exercise [53]. We hypothesize that the RSA component which usually causes cardio-respiratory coherence is disrupted due to added mental workload [58] making these features important for distinguishing between the two states. The inter-scale ordinal distance feature also shows significance for all three physical activity levels hinting at non-linear interaction between the different frequency regions. The presence of features from the dRR series show the importance of complementary non-linear information present in the series which should be further investigated. Finally, the importance of generalized entropy features calculated on the volatility series hints at the multifractal characteristics holding vital information regarding mental workload. The link between generalized entropy and multifractal heart rate characteristics has been hypothesized in [24].

## 4. Conclusions

Traditional time and frequency HRV methods don’t account for processes occurring at multiple time scales leading to complex behavior of heart rate at multiple scales. In this study, we explored the use of several multi-scale features for mental workload assessment for ambulatory users. We show that the multi-scale entropy features are robust to changing heart rate dynamics due to movement and activity and do better than the benchmark statistical and frequency domain features while also providing complementary information. This hints at the physiological processes at various time scales being effected by mental workload.

A number of different multi-scale entropy methods and scaling algorithms were tested and we found that, generally, composite coarse graining via a new second moment moving average scaling method, combined with the modified permutation entropy method outperformed other combinations, thus suggesting that multi-scale entropy methods specifically designed for short term time series are important for short term HRV analysis. Overall, the results reported herein suggest that increased workload can result in changes in ECG signal complexity of higher scales of the fractal RR series. These findings suggest that the long-term processes involved in heart rate regulation may be affected by mental workload changes. These findings allow for sufficiently accurate assessment of mental workload in safety-critical applications where users are ambulant, especially at moderate levels of physical activity. For future work, context aware models which can distinguish between different physical activity levels followed by mental workload classification could be developed. 

## Figures and Tables

**Table 1:** Benchmark heart rate variability (HRV) features extracted.

**Time Domain Features**
mean, standard deviation, coefficient of variation, rmsdd, pNN50, mean of 1st diff., standard deviation of absolute of 1st diff., normalized mean of absolute 1st diff
**Frequency Domain Features**
High frequency power (HF), normalized HF, Low frequency power (LF), normalized LF, very low frequency power, HF/LF

**Table 2:** Different scaling and entropy algorithms used.

Scaling Algorithms	Entropy Algorithms
Coarse graining (cg)	Sample Entropy
moving average (mavg)	Modified Permutation Entropy
second moment cg (mom)	Weighted Modified Permutation Entropy
moving average mom (mavg_mom)	
composite coarse graining (comp_cg)	

**Table 3:** Performance of fused comp_cg and mavg_mom scaling with mPE algorithm for different physical workload levels (* represents cases which perform significantly better (p<0.01) than chance).

Physical Activity Level	Acc	F1
No	0.7893 ± 0.0122 *	0.7886 ± 0.0131 *
Medium	0.7726 ± 0.0114 *	0.7701 ± 0.0111 *
High	0.6741 ± 0.0150 *	0.6698 ± 0.0164 *

**Table 4:** Benchmark performance comparison for the no physical activity condition (* represents cases which perform significantly better (p<0.01) than chance).

Feature (Nof)	Acc	F1
benchmark (15)	0.5772 ± 0.0192 *	0.4991 ± 0.0206
isod (66)	0.7838 ± 0.0137 *	0.7882 ± 0.0137 *
multi-scale entropy (48)	0.7893 ± 0.0122 *	0.7886 ± 0.0131 *
fused (129)	0.8438 ± 0.0126 *	0.8428 ± 0.0132 *

**Table 5:** Benchmark performance comparison for the medium physical activity condition (* represents cases which perform significantly better (p<0.01) than chance).

Feature (Nof)	Acc	F1
benchmark (15)	0.5318 ± 0.0169 *	0.6019 ± 0.0231 *
isod (66)	0.8189 ± 0.0133 *	0.8203 ± 0.0134 *
multi-scale entropy (48)	0.7726 ± 0.0114 *	0.7701 ± 0.0111 *
fused (129)	0.8401 ± 0.0128 *	0.8410 ± 0.0129 *

**Table 6:** Benchmark performance comparison for the high physical activity condition (* represents cases which perform significantly better (p<0.01) than chance).

Feature (Nof)	Acc	F1
benchmark (15)	0.5751 ± 0.0160 *	0.5393 ± 0.0245 *
isod (66)	0.7825 ± 0.0128 *	0.7818 ± 0.0137 *
multi-scale entropy (48)	0.6741 ± 0.0150 *	0.6698 ± 0.0164 *
fused (129)	0.8015 ± 0.0152 *	0.7987 ± 0.0156 *

**Table 7:** Most frequently occurring features in the top-20 feature pool for the no physical activity condition.

Feature Name	freq
mean of RR	99.2
$isod_{s1,s4}$ dRR	98.8
$isod_{s3,s9}$ RR	98.4
Coefficient of variation	93.2
lf/hf	82.8
std. absolute first difference RR	81.2
mean $isod_{s3,:}$ RR	80
${\left(mPE+comp\_cg\right)}_{s3}$ RR	70.4

**Table 8:** Most frequently occurring features in the top-20 feature pool for the medium physical activity condition.

Feature Name	freq
$isod_{s1,s2}$ RR	99.2
$isod_{s1,s7}$ RR	99.2
$isod_{s2,s10}$ RR	98
mean RR	95.2
${\left(mPE+mavg\_mom\right)}_{s1}$ RR	91.2
${\left(mPE+comp\_cg\right)}_{s9}$ RR	89.6
${\left(mPE+comp\_cg\right)}_{s1}$ RR	88.8
$isod_{s1,s4}$ dRR	83.2
${\left(mPE+comp\_cg\right)}_{s10}$ RR	81.6
${\left(mPE+mavg\_mom\right)}_{s8}$ dRR	74.4
$isod_{s2,s5}$ RR	74.4
${\left(mPE+mavg\_mom\right)}_{s8}$ RR	70.4

**Table 9:** Most frequently occurring features in the top-20 feature pool for the high physical activity condition.

Feature Name	freq
mean abs. first difference RR	100
${\left(mPE+comp\_cg\right)}_{s8}$ RR	96
lfnu	95.2
${\left(mPE_{wt}+mavg\_mom\right)}_{s3}$ dRR	93.6
hfnu	90.8
${\left(mPE+comp\_cg\right)}_{s4}$ RR	90.8
$isod_{s3,s4}$ RR	84.8
$isod_{s2,s9}$ RR	79.6
${\left(mPE+comp\_cg\right)}_{s6}$ dRR	79.6
${\left(mPE+comp\_cg\right)}_{s2}$ dRR	70.8

## Abbreviations

The following abbreviations are used in this manuscript:

RSA	Respiratory sinus arrhythmia
RR	inter-beat interval series
dRR	Absolute difference inter-beat interval series
ANS	Autonomic nervous system
NASA-TLX	NASA task load index
SWAT	Subjective workload assessment technique
ECG	Electrocardiogram
MSE	Multi-scale entropy
PE	Permutation entropy
mPE	Modified permutation entropy
HRV	Heart rate variability
MATB	Multi-attribute task battery
